# Theodore Brown Rasmussen (1910–2002)

**DOI:** 10.1007/s00415-013-6862-x

**Published:** 2013-02-21

**Authors:** Frank W. Stahnisch, Amy S. Nakashima

**Affiliations:** Departments of Community Health Sciences and History, Hotchkiss Brain Institute and Institute for Public Health, University of Calgary, 3280 Hospital Drive N.W., Calgary, AB T2N 4Z6 Canada

**Keywords:** Calgary, Epilepsy therapy, Hemispherectomy, History of medicine, Montreal, Theodore Rasmussen, 20th century

## Abstract

Dr. Theodore Rasmussen was a Canadian neurosurgeon, neurologist, and neuropathologist who primarily specialized in the treatment and histopathology of epilepsy. In addition to his eponym, “Rasmussen syndrome”, a chronic form of encephalitis accompanied by epilepsy, his contributions included the refinement of neurosurgical techniques, thoroughly cataloguing outcomes following surgery for epilepsy, and cortical mapping studies conducted with Dr. Wilder Penfield.

 In the history of medicine, innovations are often accompanied by great curiosity, along with strong criticisms; such phenomena can likewise be seen as antecedents of revolutionary change in clinical practice and medical theory [10]. This was the case with the Canadian neurological surgeon Dr. Theodore Rasmussen (1910–2002), who specialized in neurological and surgical treatments for epilepsy [3]. While Rasmussen is associated with “Rasmussen’s Syndrome” [1], a pediatric condition he characterized, in which chronic encephalitis is accompanied by epileptic seizures, his contributions to neuroscience extend well beyond this eponymous disease.

Rasmussen was born in Provo, Utah, the son of Andrew T. Rasmussen (1883–1955), who later became a neuroanatomy professor at the University of Minnesota [5]. Theodore Rasmussen received his medical training at that same university, where he graduated in 1934, before beginning a two-year internship at Kings County Hospital in Brooklyn. Between 1936 and 1939, he was a Fellow in Neurology at the innovative Medical Faculty of the Mayo Clinic in Rochester, Minnesota, continuing his neurosurgical training between 1939 and 1942 at the Montreal Neurological Institute (MNI) in Canada under the eminent neurosurgeons Wilder Graves Penfield (1891–1976), William Cone (1897–1959) and Arthur Elvidge (1899–1985). He received an M.Sc. research degree in neurology in 1939, but after completion of his postgraduate training Rasmussen was conscripted into the US Army Medical Corps. He was made the chief of the neurosurgical section of the 14th Evacuation Hospital on the Ledo Road in the China–India–Burma war zone. He was not discharged until 1945, after which he returned to Canada and became a Clinical Lecturer in Neurology and Neurosurgery at McGill University. Only two years later, Rasmussen received a Professorship in Neurological Surgery at the University of Chicago and married his Canadian fiancée, Catherine Archibald (1921–1998). They lived in Chicago until 1954, when Rasmussen returned to Montreal as a professor at McGill, while also serving as Neurosurgeon-in-Chief at the Royal Vic (Victoria Hospital). He later went on to succeed Penfield as the director of the MNI, serving from 1960 to 1972 [4].

Surgical treatment options for epilepsy had been markedly advanced in Germany by the Breslau neurologist Otfrid Foerster (1873–1941), with whom Penfield had learned his operative techniques during two research periods in 1928 and 1931. Upon returning from Europe, Penfield successfully developed a similar treatment centre in Montreal, where patients’ cortices were neurophysiologically mapped and stimulated in intraoperative settings. Together with his mentor Penfield and a number of German-speaking émigré neuroscientists, among whom were Jerzy Olszewski (1913–1964) and Fred Andermann (b. 1930), Rasmussen succeeded in further developing neurological and operative treatment options for epilepsy [9]. As a most versatile neurological surgeon, he later pioneered hemispherectomy as a curative therapy to suppress the cortical spread of pathologic activity in intractable epilepsy. This option became used in children whose brains were sufficiently plastic to adapt to the loss of half the cortex, allowing physiological functions to be relearned even after such a drastic operation [7].

Rasmussen’s collaboration with Penfield culminated in the seminal work, “The Cerebral Cortex of Man”, in which their intricate electrostimulatory and neurosurgical findings were published [6]. Together with the Japanese–Canadian physician Juhn Atsuhi Wada (b. 1926), he further introduced intracarotid amobarbital tests for the functional lateralization of speech and memory, a research approach that was complementary with Rasmussen’s efforts to preserve function when removing damaged neural tissue in epileptic patients. In addition, he was a pioneer in pituitary gland surgery and also performed innovative operations in patients with cerebral and spinal tumours. Through his compilations of large data-sets that documented the outcome of epilepsy surgery, Rasmussen explored the efficiency of his surgical techniques, while also characterizing the histopathology of chronic encephalitis in several conditions:

“We compared 100 patients with temporal lobe epilepsy, who exhibited the running down phenomenon (i.e. postoperative seizures that eventually remit following a period of months to years) following temporal resections, with two groups of patients (…). We found a significant correlation between prognosis and the size of the epileptogenic area as defined” [8].

Rasmussen’s thorough epidemiological research program, which examined the plastic adaptation of the human cortex after removal of epileptic tissue, sustained his impact on medicine beyond his time as an active neurosurgeon and director of the MNI, while laying the basis for his legacy as an outstanding investigator and teacher in clinical neuroscience. During the first three postwar decades, Rasmussen had probably performed more operations for epilepsy than most contemporary neurosurgeons. He was a member of The Society of Neurological Surgeons since 1950, its president in 1970, and was eventually honoured with the Society’s Distinguished Service Award in 1989. A number of his trainees later went on to head neurosurgery departments throughout North America. As a private man, Rasmussen was also a musician, playing both the clarinet and saxophone, and had used these talents already to supplement his allowance in his early medical school days. His colleagues and students warmly remember Rasmussen for his social amenability and educational talents:

“He was always precise and deliberate; rounds were conducted at a half-trot. His aphorisms became part of the mental fabric of all his trainees. ‘Must not do that. Do not jiggle the brain,’ and ‘Put up the side rails; the bed is high and the floor is hard,’ spring to mind. While not effusive in his manner, he was never unkind or sarcastic” [2].

After the death of his wife in 1998, he relocated westward from Montreal in Quebec to be with his children in Calgary, Alberta, where he was frequently approached by members of the Calgary Pathology Department who asked for advice on histopathological specimens from patients with severe epilepsy and brain tumours. Theodore Rasmussen passed away in 2002.
